# Supplementary material for: From sequence to enzyme mechanism using multi-label machine learning
Source: BMC Bioinformatics. 2014 May 19;15:150. doi: 10.1186/1471-2105-15-150 (PMC4229970; doi:10.1186/1471-2105-15-150)
Supplement: Additional file 2 — Java code of ml2db. Additional file ml2db_code.tar.gz contains the Java source code to run the multi-label machine learning experiments and save the results to database. The code’s Javadoc is included. [file 1471-2105-15-150-S2.zip › additional file 2/ml2db/ml2db/doc/main/ml2db/graphviz/GraphViz.html]

GraphViz


JavaScript is disabled on your browser.


- Overview
- Package
- Class
- Use
- Tree
- Deprecated
- Index
- Help

- Prev Class
- Next Class

- Frames
- No Frames

- All Classes

- Summary:
- Nested |
- Field |
- Constr |
- Method

- Detail:
- Field |
- Constr |
- Method


main.ml2db.graphviz

## Class GraphViz

- java.lang.Object
- - main.ml2db.graphviz.GraphViz

- Direct Known Subclasses:
  :   GraphVizArff

  ---

    

  ```
  public class GraphViz
  extends java.lang.Object
  ```

  Purpose: GraphViz Java API: Description:: With this Java class you can simply call dot from your Java programs Example usage:: ``` GraphViz gv = new GraphViz(); gv.addln(gv.start_graph()); gv.addln("A -> B;"); gv.addln("A -> C;"); gv.addln(gv.end_graph()); System.out.println(gv.getDotSource()); String type = "gif"; File out = new File("out." + type); // out.gif in this example gv.writeGraphToFile(gv.getGraph(gv.getDotSource(), type), out); ```

  Version:
  :   v0.4, 2011/02/05 (February) -- Patch of Keheliya Gallaba is added.
      Now you can specify the type of the output file: gif, dot, fig, pdf,
      ps, svg, png, etc., v0.3, 2010/11/29 (November) -- Windows support + ability to read the
      graph from a text file, v0.2, 2010/07/22 (July) -- bug fix, v0.1, 2003/12/04 (December) -- first release

  Author:
  :   Laszlo Szathmary (jabba.laci@gmail.com)

- - ### Field Summary

    Fields

    | Modifier and Type | Field and Description |
    | --- | --- |
    | `static java.lang.String` | `DOT` |
    | `static java.lang.String` | `DOT_COMMENT_PREFIX` |
    | `static java.lang.String` | `DOT_UNDIRECTED_EDGE` |
    | `static java.lang.String` | `FIG` |
    | `static java.lang.String` | `GIF` |
    | `static java.lang.String` | `JPG` |
    | `static java.lang.String` | `PDF` |
    | `static java.lang.String` | `PLAIN` |
    | `static java.lang.String` | `PNG` |
    | `static java.lang.String` | `PS` |
    | `static java.lang.String` | `SVG` |
  - ### Constructor Summary

    Constructors

    Constructor and Description

    `GraphViz()`

    Constructor: creates a new GraphViz object that will contain a graph.

    `GraphViz(java.lang.String layoutAlgorithm)`

    Constructor: creates a new GraphViz object that will contain a graph.
  - ### Method Summary

    Methods

    | Modifier and Type | Method and Description |
    | --- | --- |
    | `void` | `add(java.lang.String line)` Adds a string to the graph's source (without newline). |
    | `void` | `addln()` Adds a newline to the graph's source. |
    | `void` | `addln(java.lang.String line)` Adds a string to the graph's source (with newline). |
    | `java.lang.String` | `end_graph()` Returns a string that is used to end a graph. |
    | `java.lang.String` | `getDotSource()` Returns the graph's source description in dot language. |
    | `byte[]` | `getGraphImage(java.lang.String dot_source, java.lang.String type)` Returns the graph as an image in binary format. |
    | `void` | `readSource(java.lang.String input)` Read a DOT graph from a text file. |
    | `java.lang.String` | `start_graph()` Returns a string that is used to start a graph. |
    | `java.lang.String` | `toGif(java.lang.String fileName, java.lang.String directory)` Writes this object dot graph to GIF image in the desired directory |
    | `java.lang.String` | `toGifToTmp(java.lang.String fileName)` Writes this object dot graph to GIF image in the \tmp\ directory |
    | `java.lang.String` | `toImage(java.lang.String fileName, java.lang.String directory, java.lang.String imageFormat)` Writes this object dot graph to the desired image format |
    | `void` | `toImageFile(java.lang.String type, java.lang.String filePath)` Writes this object dot code to image file |
    | `java.lang.String` | `toJpg(java.lang.String fileName, java.lang.String directory)` Writes this object dot graph to GIF image in the desired directory |
    | `java.lang.String` | `toPdf(java.lang.String fileName, java.lang.String directory)` |
    | `java.lang.String` | `writeDotSourceToFile(java.lang.String filePath)` Writes the source of the graph in a file, and returns the written file as a File object. |
    | `int` | `writeGraphToFile(byte[] img, java.io.File to)` Writes the graph's image in a file. |
    | `int` | `writeGraphToFile(byte[] img, java.lang.String file)` Writes the graph's image in a file. |

    - ### Methods inherited from class java.lang.Object

      `equals, getClass, hashCode, notify, notifyAll, toString, wait, wait, wait`

- - ### Field Detail


    - #### GIF

      ```
      public static final java.lang.String GIF
      ```

      See Also:
      :   Constant Field Values


    - #### JPG

      ```
      public static final java.lang.String JPG
      ```

      See Also:
      :   Constant Field Values


    - #### DOT

      ```
      public static final java.lang.String DOT
      ```

      See Also:
      :   Constant Field Values


    - #### FIG

      ```
      public static final java.lang.String FIG
      ```

      See Also:
      :   Constant Field Values


    - #### PDF

      ```
      public static final java.lang.String PDF
      ```

      See Also:
      :   Constant Field Values


    - #### PS

      ```
      public static final java.lang.String PS
      ```

      See Also:
      :   Constant Field Values


    - #### SVG

      ```
      public static final java.lang.String SVG
      ```

      See Also:
      :   Constant Field Values


    - #### PNG

      ```
      public static final java.lang.String PNG
      ```

      See Also:
      :   Constant Field Values


    - #### PLAIN

      ```
      public static final java.lang.String PLAIN
      ```

      See Also:
      :   Constant Field Values


    - #### DOT\_UNDIRECTED\_EDGE

      ```
      public static final java.lang.String DOT_UNDIRECTED_EDGE
      ```

      See Also:
      :   Constant Field Values


    - #### DOT\_COMMENT\_PREFIX

      ```
      public static final java.lang.String DOT_COMMENT_PREFIX
      ```

      See Also:
      :   Constant Field Values
  - ### Constructor Detail


    - #### GraphViz

      ```
      public GraphViz()
      ```

      Constructor: creates a new GraphViz object that will contain a graph.


    - #### GraphViz

      ```
      public GraphViz(java.lang.String layoutAlgorithm)
      ```

      Constructor: creates a new GraphViz object that will contain a graph.
  - ### Method Detail


    - #### add

      ```
      public void add(java.lang.String line)
      ```

      Adds a string to the graph's source (without newline).


    - #### addln

      ```
      public void addln()
      ```

      Adds a newline to the graph's source.


    - #### addln

      ```
      public void addln(java.lang.String line)
      ```

      Adds a string to the graph's source (with newline).


    - #### end\_graph

      ```
      public java.lang.String end_graph()
      ```

      Returns a string that is used to end a graph.

      Returns:
      :   A string to close a graph.


    - #### getDotSource

      ```
      public java.lang.String getDotSource()
      ```

      Returns the graph's source description in dot language.

      Returns:
      :   Source of the graph in dot language.


    - #### getGraphImage

      ```
      public byte[] getGraphImage(java.lang.String dot_source,
                         java.lang.String type)
      ```

      Returns the graph as an image in binary format.

      Parameters:
      :   `dot_source` - Source of the graph to be drawn.
      :   `type` - Type of the output image to be produced, e.g.: gif, dot, fig,
          pdf, ps, svg, png.

      Returns:
      :   A byte array containing the image of the graph.


    - #### readSource

      ```
      public void readSource(java.lang.String input)
      ```

      Read a DOT graph from a text file.

      Parameters:
      :   `input` - Input text file containing the DOT graph source.


    - #### start\_graph

      ```
      public java.lang.String start_graph()
      ```

      Returns a string that is used to start a graph.

      Returns:
      :   A string to open a graph.


    - #### toGif

      ```
      public java.lang.String toGif(java.lang.String fileName,
                           java.lang.String directory)
      ```

      Writes this object dot graph to GIF image in the desired directory

      Parameters:
      :   `fileName` - the desired file name for the image (the file type is added
          automatically)
      :   `directory` - the directory path

      Returns:
      :   the written file full path or null if the file cannot be read


    - #### toGifToTmp

      ```
      public java.lang.String toGifToTmp(java.lang.String fileName)
      ```

      Writes this object dot graph to GIF image in the \tmp\ directory

      Parameters:
      :   `fileName` - the desired file name for the image (the \tmp\ path and file
          type are added automatically)

      Returns:
      :   the written file full path or null if the file cannot be read


    - #### toImage

      ```
      public java.lang.String toImage(java.lang.String fileName,
                             java.lang.String directory,
                             java.lang.String imageFormat)
      ```

      Writes this object dot graph to the desired image format

      Parameters:
      :   `fileName` - the desired file name for the image (the file type is added
          automatically)
      :   `directory` - the directory path

      Returns:
      :   the written file full path or null if the file cannot be read


    - #### toImageFile

      ```
      public void toImageFile(java.lang.String type,
                     java.lang.String filePath)
      ```

      Writes this object dot code to image file

      Parameters:
      :   `type` - the image type (jpg, gif, pdf, etc.)
      :   `filePath` - the desired file path and file name for the image, without the
          dot and file type.


    - #### toJpg

      ```
      public java.lang.String toJpg(java.lang.String fileName,
                           java.lang.String directory)
      ```

      Writes this object dot graph to GIF image in the desired directory

      Parameters:
      :   `fileName` - the desired file name for the image (the file type is added
          automatically)
      :   `directory` - the directory path

      Returns:
      :   the written file full path or null if the file cannot be read


    - #### toPdf

      ```
      public java.lang.String toPdf(java.lang.String fileName,
                           java.lang.String directory)
      ```


    - #### writeDotSourceToFile

      ```
      public java.lang.String writeDotSourceToFile(java.lang.String filePath)
      ```

      Writes the source of the graph in a file, and returns the written file as
      a File object.

      Parameters:
      :   `filePath` - the directory + file name where the .dot file should be saved

      Returns:
      :   The file path that contains the source of the graph.


    - #### writeGraphToFile

      ```
      public int writeGraphToFile(byte[] img,
                         java.io.File to)
      ```

      Writes the graph's image in a file.

      Parameters:
      :   `img` - A byte array containing the image of the graph.
      :   `to` - A File object to where we want to write.

      Returns:
      :   Success: 1, Failure: -1


    - #### writeGraphToFile

      ```
      public int writeGraphToFile(byte[] img,
                         java.lang.String file)
      ```

      Writes the graph's image in a file.

      Parameters:
      :   `img` - A byte array containing the image of the graph.
      :   `file` - Name of the file to where we want to write.

      Returns:
      :   Success: 1, Failure: -1


- Overview
- Package
- Class
- Use
- Tree
- Deprecated
- Index
- Help

- Prev Class
- Next Class

- Frames
- No Frames

- All Classes

- Summary:
- Nested |
- Field |
- Constr |
- Method

- Detail:
- Field |
- Constr |
- Method
